# Multiple-level stakeholder engagement in malaria clinical trials: addressing the challenges of conducting clinical research in resource-limited settings

**DOI:** 10.1186/s13063-018-2563-1

**Published:** 2018-03-22

**Authors:** George Mtove, Joshua Kimani, William Kisinza, Geofrey Makenga, Peter Mangesho, Stephan Duparc, Miriam Nakalembe, Kamija S. Phiri, Russell Orrico, Ricardo Rojo, Pol Vandenbroucke

**Affiliations:** 1 National Institute for Medical Research, Amani Medical Research Centre, Muheza, Tanzania; 2 College of Health Sciences, University of Nairobi, Nairobi, Kenya; 3 Medicines for Malaria Venture, Geneva, Switzerland; 4 School of Medicine, Makerere University, Kampala, Uganda; 5 College of Medicine, University of Malawi, Blantyre, Malawi; 6 Pfizer, Collegeville, PA USA; 7 Pfizer, Groton, CT USA; 8 Pfizer, New York, NY USA

**Keywords:** Malaria, Community engagement, Local customs, Resource-limited settings, Clinical trials

## Abstract

**Background:**

Multinational clinical trials are logistically complex and require close coordination between various stakeholders. They must comply with global clinical standards and are accountable to multiple regulatory and ethical bodies. In resource-limited settings, it is challenging to understand how to apply global clinical standards to international, national, and local factors in clinical trials, making multiple-level stakeholder engagement an important element in the successful conduct of these clinical trials.

**Main body:**

During the planning and implementation of a large multinational clinical trial for intermittent preventive treatment of malaria in pregnancy in resource-limited areas of sub-Saharan Africa, we encountered numerous challenges, which required implementation of a range of engagement measures to ensure compliance with global clinical and regulatory standards. These challenges included coordination with ongoing global malaria efforts, heterogeneity in national regulatory structures, sub-optimal healthcare infrastructure, local practices and beliefs, and perspectives that view healthcare providers with undue trust or suspicion.

In addition to engagement with international bodies, such as the World Health Organization, the Malaria in Pregnancy Consortium, the Steve Biko Centre for Bioethics, and the London School of Hygiene and Tropical Medicine, in order to address the challenges just described, Pfizer Inc. and Medicines for Malaria Venture (the “Sponsoring Entities” for these studies) and investigators liaised with national- and district-level stakeholders such as health ministers and regional/local community health workers. Community engagement measures undertaken by investigators included local meetings with community leaders to explain the research aims and answer questions and concerns voiced by the community. The investigators also engaged with family members of prospective trial participants in order to be sensitive to local practices and beliefs.

**Conclusion:**

Engagement with key stakeholders at international and national levels enabled the Sponsoring Entities to address challenges by aligning the study design with the requirements of health and regulatory agencies and to understand and address healthcare infrastructure needs prior to trial initiation. Local stakeholder engagement, including community members, study participants, and family enabled the investigators to address challenges by ensuring that study design and conduct were adapted to local considerations and ensuring accurate information about the study aims was shared with the public.

**Trial registration:**

ClinicalTrials.gov, ID: NCT01103063. Registered on 7 April 2010.

## Background

Multinational clinical trials are logistically complex and require close coordination between stakeholders with varying areas of expertise. They require detailed planning by “Sponsoring Entities” and engagement with international health-policy bodies and national- and district-level stakeholders such as health ministers and regional/local community health workers. These clinical trials must comply with global clinical standards and are accountable to multiple regulatory and ethical bodies. In resource-limited settings, the challenges of running a clinical trial are considerably greater, due to infrastructure that is sub-optimal to meet the basic needs of the community or to support a clinical trial (limited access to healthcare personnel and limited physical infrastructure is often exacerbated by high burdens of underlying diseases of poverty) and barriers to community and patient participation due to practices and beliefs that may challenge study designs or view healthcare providers (and by extension, clinical trial participation) with undue trust or undue suspicion [1]. These factors can make it difficult to understand how to apply global clinical standards in resource-limited settings and require community engagement by study teams.

### Regulatory considerations

National regulatory agencies may lack the capacity, expertise, or experience required to review and approve protocols for complex clinical trials, especially in situations where changes at the ministerial level in the Ministry of Health lead to reorganization of the regulatory agency and an influx of new, inexperienced staff. Therefore, it is important to ensure that national regulatory agencies meet World Health Organization (WHO) criteria for effective regulatory authority.

### Infrastructure considerations

Limited healthcare infrastructure, socioeconomic disadvantage, illiteracy, poor general health, or unfamiliarity with the scientific rationale behind medical research in general, can all represent substantial barriers to an individual’s agreement and proper consent to participate in a clinical trial. To overcome some of these barriers, it is essential to engage key stakeholders at all levels of the healthcare system. These stakeholders include the following: international organizations, non-governmental aid agencies, national policy-makers, decision-makers at the district level (e.g., district administrative officers and Council Health Management Teams (CHMTs)), local community leaders, local community members with relevant expertise (e.g., teachers, healthcare workers, civil servants, agricultural officers, and religious and traditional leaders), families, and individual patients.

### Bioethics considerations

There are specific ethical challenges in running a clinical trial in resource-limited settings that may differ from those encountered in more developed areas [2]. These include challenges associated with limited healthcare infrastructure; practices and beliefs, including patient autonomy; and perceptions of healthcare providers and institutions.

Access to adequate healthcare is often limited or prohibitively expensive, so the chance to receive free healthcare as part of trial participation could influence the decision to participate. Thus, the choice to participate, by presenting alternatives that are “out of reach” of the participant, may appear to not offer an alternative [3–7]. As such, there must be a clear effort to ensure that consent to trial participation is fully informed and not unduly influenced by the prospect of receiving free healthcare. An additional challenge presented by limited healthcare infrastructure is the implications this has for Sponsoring Entities’ ability to outfit the research area in a way that can provide for subject safety and also benefit the communities. Additionally, ethics committees in resource-limited settings are often over-stretched, under-staffed, or may lack some of the expertise that is accepted as standard in developed countries. All of these factors can combine to create substantial challenges. The study design, implementation, and informed consent process must, therefore, be prepared with an understanding of these considerations.

Contemporary Western health psychology and health educational frameworks that consider “the individual” as the foundation for decision-making may be incompatible in the developing world, where some authors have identified communalism or “social autonomy” to be the norm [8]. As a result, community opinion, or advice from persons of influence within the community, may influence the individual’s decision to join a trial. This constitutes another, slightly different consideration for the informed consent process wherein the community must be engaged and public perceptions of the research must be actively managed. Family members may also need to be involved in the process. Additional local practices and beliefs must be understood and accounted for in study design and conduct. Respect for these culturally distinguishing features of a community must be on the forefront of the researcher’s planning.

Perceptions of healthcare professionals or institutions must also be understood and accounted for. Perceived power differentials between physicians and patients may be a source of ethical challenges in the informed consent process and throughout study conduct. Education and clarification of research aims must be undertaken in this setting.

### Multiple-level stakeholder engagement

This article describes the stakeholder engagement efforts undertaken by the investigators and Pfizer Inc. and Medicines for Malaria Venture (the “Sponsoring Entities” for these trials) in planning and running a large, multicenter clinical trial evaluating intermittent preventive treatment of malaria in pregnancy (IPTp) in five countries in sub-Saharan Africa using azithromycin-chloroquine (AZCQ) versus sulfadoxine-pyrimethamine (NCT01103063) [9] (see also Table 1). The lessons learned apply not only to this particular trial but also to the conduct of clinical trials in resource-constrained areas in general.

**Table 1:** Overview of the study

Study title	A Phase 3, Open-label, Randomized, Comparative Study to Evaluate Azithromycin plus Chloroquine and Sulfadoxine plus Pyrimethamine Combinations for Intermittent Preventive Treatment of Falciparum Malaria Infection in Pregnant Women in Africa
ClinicalTrial.gov identifier	NCT01103063
Primary objective	The primary objective was to establish superiority of AZCQ over SP in protective efficacy for intermittent preventative treatment in pregnancy (IPTp) as measured by the proportion of subjects with sub-optimal pregnancy outcome defined as any of the following: low-birth-weight live birth, premature birth, abortion, still birth, lost to follow-up prior to delivery or termination of pregnancy, or missing birth weight of the neonate
Key secondary objectives	1. Proportion of subjects with low-birth-weight live neonates 2. Proportion of subjects with severe anemia 3. Proportion of subjects with anemia 4. Proportion of subjects with placental parasitemia 5. Occurrence of sexually transmitted infections 6. Safety and tolerability of the two treatment regimens 7. Presence of subjects with a sub-optimal pregnancy outcome including those characteristics described in the primary outcome with the addition of neonatal deaths and congenital malformations
Study sites	Cotonou, Benin; Siaya, Kenya; Zomba, Malawi; Muheza, Tanga, Tanzania; Mwanza, Tanzania; and Kampala, Uganda
Subjects	Pregnant women age 16 years to 35 years
Study drugs	AZCQ 250 mg/155 mg QD for 3 days (3 treatments at 4–8-week intervals)
	SP 500 mg/25 mg QD (3 treatments at 4–8-week intervals)

*AZCQ* azithromycin/chloroquine, *QD* four times a day***,*** *SP* sulfadoxine-pyrimethamine

### At the international level

**Fig. 1:**
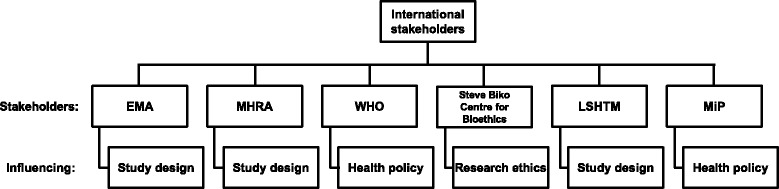
International stakeholders. EMA, European Medicines Agency; MHRA, UK Medicines and Healthcare products Regulatory Agency; WHO, World Health Organization; LSHTM, London School of Hygeine and Tropical Medicine; MiP, Malaria in Pregnancy consortium

Effective malaria control and eradication requires collaborative and concerted efforts that go beyond national boundaries (Fig. 1). Any clinical trial on an existing or novel malaria treatment should be undertaken with knowledge and awareness of ongoing initiatives by various international organizations involved in the global fight against malaria. The WHO plays a major role in malaria control and eradication strategies worldwide and collaborates with partners in the public, private, and non-profit sectors [10]. The WHO-Global Malaria Program (WHO-GMP) is the major global policy-maker in the field of the treatment of malaria, vector control, and other initiatives, and publishes expert guidelines and advice on the implementation of effective treatment and eradication strategies [11].

WHO-GMP and Ministries of Health in malaria-endemic countries have developed guidelines that recommend IPTp with sulfadoxine-pyrimethamine in areas of high malaria transmission [11]. Our trial was designed to assess the safety and efficacy of a novel combination therapy of azithromycin-chloroquine that had not at that point been studied as an option for the IPTp indication, although it had been studied for the treatment of symptomatic malaria in both adults and children in previous clinical trials [12, 13]. The study was conducted at six investigator sites in five countries in sub-Saharan Africa: Benin, Kenya, Malawi, Tanzania, and Uganda (see Table 1).

Trial design took into account changing circumstances at the international, national, and local levels to adapt to conditions that could not be foreseen at the early trial design stage. Early in the design stage of the trial, it was imperative to consider not only the current WHO-GMP malaria treatment guidelines at that time, but also to consider ongoing initiatives that would produce changes to future guidelines. For these reasons, discussions and interactions with the WHO-GMP were important in informing aspects of trial design and potentially future-proofing the trial so that it would be in line with recommendations as they changed. For example, at the time of initial study design, WHO recommended a two-treatment regimen with IPTp-SP during the antenatal period. A three-treatment regimen was supported in the scientific literature, but had not yet been established as the recommended practice. We therefore adopted the three-treatment IPTp-SP regimen as a control group so that the results of the study would be clinically meaningful by the time study results would become available. An adaptation to local practice was the use of trained field workers who verified treatment compliance, sought participants who missed visits, and performed postnatal home visits. Trial design was, therefore, a collaborative effort in which the Sponsoring Entities consulted with international and national agencies and investigators. For example, advice was sought from independent experts at institutes such as the London School of Hygiene and Tropical Medicine. Our trial was sponsored by Pfizer Inc. and the Medicines for Malaria Venture (MMV), a not-for profit organization with a stated mission to advance the cause of malaria eradication through the effective deployment of research resources (https://www.mmv.org). The expertise and experience MMV brought to the endeavor was invaluable in ensuring effective stakeholder engagement, along with the WHO-GMP and the Malaria in Pregnancy (MiP) Consortium, as well as a robust scientific knowledge base and advice and ethics committee review. The WHO-GMP was also vital in advising national authorities on regulatory review procedures.

An important consideration in conducting research in resource-limited settings is the fair distribution of benefits [14–17]. It is vital that capacity building in preparation for the clinical trial confers a benefit to the community involved in the research and that any new treatment that may result from the research is accessible and affordable in the target countries. To this end, we engaged with a variety of global agencies to determine access. These discussions assured the Sponsoring Entities that there was a high probability that financial support mechanisms that would make the proposed treatment accessible and affordable in the areas of need would be in place. Additional efforts regarding fair distribution of benefits were undertaken, but are beyond the scope of this article and have been discussed in general by others [18, 19].

Alignment with the needs of regulators and policy-makers was achieved through interactions with organizations such as the European Medicines Agency (EMA), the UK Medicines and Healthcare Products Regulatory Agency (MHRA), the Steve Biko Center for Bioethics at the University of the Witwatersrand in Johannesburg, South Africa, the London School of Hygiene and Tropical Medicine, the MiP consortium, and malaria and maternal health experts in the WHO. Important points of discussion were the research aims of the study, the commitments of the Sponsoring Entities, and the plans for next steps depending on the success or failure of the study. The needs and expectations of these organizations were sought early in the design of the trial and influenced study endpoints and treatment regimens. This was vital in ensuring that the study met their expectations.

### At the national and district levels

**Fig. 2:**
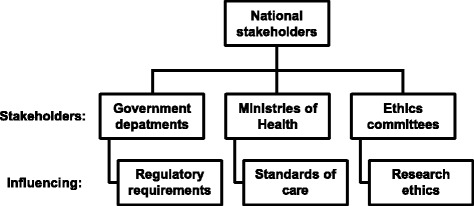
National stakeholders

At the country level, it was necessary to engage national policy-makers, including governmental departments, health ministries, and ethics committees (Fig. 2). Policies can differ from country to country with regard to regulatory issues, healthcare infrastructure, standard of care, age of majority, informed consent, and ethical approval.

In Tanzania, for example, before initiating the trial, the National Malaria Control Program (NMCP) of the Ministry of Health and Social Welfare was engaged and provided guidance with respect to malaria research and policy implications. In their initial review of the protocols, TFDA expressed concern about using a drug combination that included chloroquine due to the well-documented chloroquine-resistant malaria in Tanzania that preceded a suspension of chloroquine use for the treatment of falciparum malaria in the country. We had a successful meeting with the NMCP followed by approval from the National Health Research Ethics Committee. The Tanzania Food and Drug Authority (TFDA), which is responsible for control of clinical trials, was key to providing regulatory guidance. Whenever necessary and possible, we convened meetings between the study team and the TFDA for more explanation on their concerns regarding our application. Through correspondence and discussion with TFDA, the Sponsoring Entities were able to share information about the in vitro and in vivo efficacy of the chloroquine-containing regimen in combination with azithromycin against chloroquine-resistant *P. falciparum* and to point out the growing concern about SP resistance in the setting of prophylactic use. As a result of these interactions, TFDA approved the clinical trial applications for these studies.

The diversity of the countries involved in such a large trial ensures the strength of the findings, but it also brings additional challenges in managing different policies or interpretations of international guidelines. Effective and frequent engagement with representatives of the national health ministry, ethics committees, regulatory authority, and other national agencies was important in meeting the expectations of each country involved in the study. Information-sharing and dialog about results from previous research enabled national stakeholders to appreciate the scientific merits of the current research while helping the Sponsoring Entities to appreciate the concerns of national policy-makers and regulators.

At the interface of the national institutions and local communities are the district and divisional representatives of the Ministries of Health. These are the initial entry points to the community. They include Ministry of Health representatives, district administrative officers, and boards that oversaw all health-related issues at the district level. CHMT in Tanzania is an example of this district-level board, which is chaired by the District Medical Officer, and its members include the District Reproductive and Child Health Coordinator, the District Malaria Coordinator, the District Nurse Officer, the District Pharmacist, etc. Collaboration with individuals at this level is vital in ensuring adherence to national policies. They require to be kept apprised of the progress of the study, with regular updates on topics such as ethical and regulatory approvals. We found that these individuals were particularly interested in information on potential side effects, adverse events, or issues that had been raised by community leaders. Efficient and regular communication with these district-level stakeholders was essential in order to build trust with the local community and improve compliance with the study protocol [20–22].

### At the local community level

**Fig. 3:**
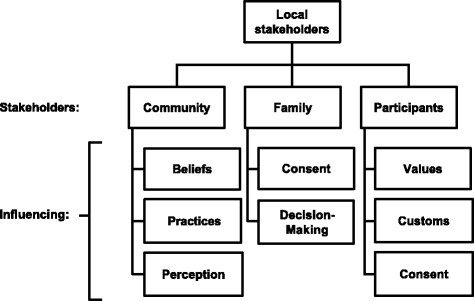
Local stakeholders

The successful implementation of our clinical trial on malaria therapy was directly dependent upon a good relationship with the local community to keep stakeholders informed of the aims and objectives of the project and the possible outcomes (Fig. 3). This was especially important in rural or semi-rural areas, where village leaders, elders, and religious leaders are often seen as the most trusted authority figures. Engagement with such individuals was an important means of gaining the trust of the local community, explaining the project goals, and describing the complexities of likely risks and benefits to prospective study participants.

As other researchers have found, a recommended method of community engagement is to have an initial briefing followed by repeated consultations and progress updates through the use of community advisory boards, the frequency of which would depend upon the level of information exchanged, stage of research, and issues or problems needing to be addressed [23, 24]. The relationship with community representatives began with consultation and eventually gave way to collaboration, as they contributed vital local knowledge on assumptions, customs, and beliefs that were pertinent to the effective operation of the trial and provided insights into how these factors might be effectively addressed. In some cases, feedback from community representatives led to adaptations and improvements to the protocol such as additional clarification on exclusion and inclusion criteria, inclusion of plans to ensure prompt follow-up, and specification of mitigation strategies to avoid loss to follow-up. Feedback from community representatives was also beneficial in communicating the expectations of the community with regard to any long-lasting benefits accruing from the study, such as improvements to healthcare infrastructure through up-front investment in capacity building efforts of the Sponsoring Entities.

Development of drugs intended for pregnant women in certain resource-limited settings can present additional challenges for researchers. There may be beliefs or practices with respect to pregnancy and delivery that may need to be taken into account. For example, while our protocol required placental or cord blood samples, it has to be understood that in some communities there are long-standing traditions or beliefs about the handling of these tissues; the reasons for the requirements for these samples must be clearly explained to potential participants while not failing to demonstrate appropriate respect for traditions and beliefs. In some countries, the placenta is treated with a great deal of reverence. Placental burial traditions are common in many resource-limited settings [25]. It is thus important to reassure local communities and individual study participants that placental tissue will be treated respectfully.

Local community relationships were also vital in understanding and addressing myths or misconceptions that had the potential to undermine trust in the aims and potential benefits of this study. For example, there was an ongoing rumor in Tanzania, reported widely in the national press, of involvement of secretive fraternal organizations in various government-funded and externally funded research programs. These rumors included one of the investigator sites participating in our study. It is important to note that the existence of rumors such as these are a relatively common phenomenon when undertaking research in resource-limited areas and they often represent a manifestation of underlying concerns about the impact or effects of study participation that are quite distinct from the rumor itself; thus, they can be considered to be “metaphors” by which the local community express their concerns in a way that is culturally familiar to them [26, 27]. To mitigate the potential effects of these rumors to undermine our ability to conduct this research, we held meetings with the CHMTs and the study team in an effort to engage the community. The CHMTs helped us as we sensitized the community, including its leaders, in order to explain the scope and objectives of the study and its likely impact on the community. This was a continuous process, with regular meetings to clarify issues and provide updates. This approach was instrumental in gaining and maintaining the trust of the local community.

Local stakeholders within the national research organizations and hospitals (including health centers and dispensaries) are essential partners in ensuring adequate infrastructure and healthcare delivery that is in line with local guidelines. Community health workers are the link between the local community and healthcare systems, ensuring that mothers attend ante-natal clinics and comply with follow-up visits. This role is vital given the anecdotal evidence that in some countries, women are reluctant to attend ante-natal clinics due to the stigma that these clinics are associated with human immunodeficiency virus (HIV) treatment or compulsory HIV testing, and in some cases have a reputation for a lack of empathy with respect to the women receiving care. Community health workers ensured high rates of compliance with treatment by direct supervision of study drug administration. They were also vital in ensuring the use of insecticide-treated bednets, which were provided as part of the study implementation and were used successfully, with high compliance rates. In Tanzania, plans are underway to formally incorporate community health workers into the health infrastructure, thereby further solidifying links with local communities and the national healthcare system [15–17]. Local contacts are integral to building local operational capacity, organizing what can sometimes be rather limited resources, and providing valuable feedback to the Sponsoring Entities on areas that may need additional support or investment. It was also deemed necessary to ensure strong local or regional representation on an independent external data monitoring committee.

Training and certification of laboratory staff members and microscopists was carried out by independent experts. The Sponsoring Entities helped to build sustainable site infrastructure as necessary, including such elements as providing laboratory equipment and training and providing additional short- and long-term file storage. The Sponsoring Entities also implemented intensive training in Good Clinical Practice (GCP), protocol implementation, and bioethics for the investigator site study teams. Bioethics training was done in collaboration with the Steve Biko Center for Bioethics, which helped us host bioethics workshops for investigators, ethics committee members, and regulators. The Research Bioethics course examined topics such as what constitutes unethical research, relevant national and international regulations and guidelines for research ethics, protocol reviews, case studies and standards of care in a study, and authorship guidelines and plagiarism. Specific topics included obtaining valid informed consent considering specific subgroups, standards of care for trial participants, access to study medications following completion of a clinical trial, issues pertaining to incentives affecting researchers and participants, releasing and publishing research results and the implementation of research findings [28]. Training was also given to field workers who undertook home visits at each site to ensure proper safety and compliance monitoring, proper use and distribution of study medication, continuous use of insecticide-treated bednets, and correct follow-up.

Importantly, there may be substantial effort required on the part of investigator sites to support a large, multicenter pharma industry-sponsored trial aiming to support a stringent regulatory agency submission that may present difficulties not encountered in implementing academic studies that may be smaller in size and complexity with respect to GCP. For example, the volume of documentation required for a submission to regulatory authorities may be much larger than that required in academic studies. The industry Sponsoring Entity is obligated by global clinical trial guidance to adhere to their standard operating procedures and systems in the execution of clinical trials. By default, therefore, the investigational center participating in the trial becomes subject to these same standards and systems. While these requirements enhance certain aspects of study conduct, including for example robust safety reporting, independent GCP oversight, strict data handling, and investment in sustainable infrastructure/capacity, and industry sponsorship may also introduce complexity and increased study lead-time for investigator site staff to learn new systems for data capture, safety reporting, and infrastructure preparation.

In our trial, a robust monitoring plan was implemented (including frequent monitoring visits to the investigator sites) to ensure data quality. As part of this plan there were weekly calls between principal investigators and study clinicians for close medical monitoring and to share knowledge and experience from all sites. There were also bi-weekly calls between clinicians and the monitoring team to review any site-specific clinical issues and to apply learnings and best practices across investigator sites. Protocol-specific quality review visits and site audits took place on a regular basis. Enrollment caps were implemented based on ongoing assessment of a study site’s capacity to implement the protocol in compliance with GCP. These efforts supported the Sponsoring Entities’ objectives to understand local issues, promote site capacity building, and to encourage collaboration between investigator sites.

An additional concern is that the target population may have been vulnerable to inducement to participate in research due to healthcare resource constraints and the burden of disease [29]. Therefore, special attention to patient autonomy was emphasized during the informed consent process in particular. The need to offer a high level of medical care must be undertaken in a manner that acknowledges that a risk inherent in the offer of medical care is that it may become an inducement to participate in research, as might reimbursement for food and travel. Among potential study participants, researchers may also encounter deference to healthcare professionals on the one hand or suspicion of clinical research on the other. Therefore, researchers must ensure that communities are engaged and informed of research aims and must put appropriate emphasis on patient autonomy and the voluntary nature of research participation, while bearing in mind that healthcare-related decisions often involve extended family.

### At the family level

Husbands, mothers-in-law, and other members of the extended family are important stakeholders whose concerns for the safety and well-being of their family need to be taken into account. It is vital that the families of study participants are kept fully informed of study aims and possible outcomes as well as any risks and benefits to the study participant.

The impact of family based decision-making became apparent in our study when we began to see increased attrition of enrolled participants, which investigators attributed to family members “overriding” participants’ decisions to join the study. At the beginning of the study, informed consent had been undertaken with an approach that may not have fully engaged the community or family. While this practice placed an emphasis on the autonomy of the research participant, its lack of community/family engagement clearly impacted study conduct and our ability to fully evaluate research participant data. When this was realized, the study teams undertook an informed consent process that encouraged relevant family members (e.g., husbands) to be present during the informed consent discussion and reiterated the need for patients to discuss study participation with family prior to deciding to join. While the language in the informed consent document still put an emphasis on individual decision and autonomy, in practice, the informed consent process attempted to account for the local social structures, a necessary balance that has been described by others [8, 30–32].

Husbands are not the only family members who should be involved in the consultation along with the prospective study participant. In some resource-limited settings where our study was conducted, the mother-in-law is the key decision-maker in matters related to her daughter-in-law or grandchildren. This can have a substantial bearing on the likelihood of an individual participating in the study. For example, in some communities in Benin, where the mother-in-law has a prominent role in the life of the baby, failure to consult them prior to the pregnant woman being accepted into the study could result in the pregnant woman being removed from the home if she lost the baby and had not informed the mother-in-law about her participation in the trial. This extended family involvement must be acknowledged but also balanced with concern for the autonomy of the pregnant woman.

One particular issue with respect to the family is the age of legal responsibility. The age range for our study started at 16 years, given the need for 50% primi- and secundigravidae. This brought up several local cultural and legal issues with respect to consenting minors. For example, in some of our study areas, such as Malawi and Benin, a 16-year old is considered an emancipated minor if she marries or becomes pregnant. Normally, an emancipated minor is deemed able to exercise full legal responsibility and does not require the presence of a guardian or legally authorized representative during the consent process. For the purposes of our trial, however, we still required a parent or legal guardian to be present during the consent process, as the research was situated within the context of global clinical research standards. This created logistical problems due to discrepancies between cultural norms and the requirements of our trial. All women under 18 years of age had to have a legal guardian, although in many instances, subjects under the age of 18 who were married arrived at the hospital on their own or with their husband who was also under the age of 18. This led to situations where the presence of a legal guardian was required even though local law and custom would not have such a requirement.

In addition, in some countries, it may be difficult to obtain a legally binding document to establish that someone is a legally authorized representative due to administrative issues such as the time taken by courts to issue such documents or long distances between the court and the study site.

### Challenges in obtaining informed consent

A major challenge faced in running a clinical trial in resource-limited settings is navigating language and literacy issues in obtaining informed consent. Although, English, French, and Swahili are the official languages in our research areas, subjects often preferred to be consented in their local language, which may be a predominantly spoken language with a written form or script that is unfamiliar outside of academic circles. During our trial, as part of the local site’s informed consent process, subjects were asked for their preferred language for giving consent (e.g., English, French, Swahili, Dholuo, Fon, etc.). Literacy level was then evaluated in the preferred consenting language to ensure that consent was properly understood and to determine the need for an impartial witness.

For example, in Siaya, Kenya, all participants were consented using their language of choice after confirming an acceptable literacy level. Additionally, the person consenting was required to be literate with a good level of comprehension in the selected language. Subjects who could not read or struggled to read and write in their local language were assumed to be illiterate, and the site had to use an impartial witness during the informed consent process. Subjects wishing to be consented in those languages for which there is no written form were treated in the same way as subjects who were illiterate: The site used an impartial witness in the informed consent process. It was planned that alternative methods of consenting may be used, e.g., videotaping or voice recording by a certified translator that would be played during the informed consenting process. Ultimately, these techniques were not used for logistical reasons related to implementation timelines, but they were demonstrated and explained as a contingency plan during investigator meetings. It should be noted, however, that not all ethics committees may approve such alternative methods for cultural, religious, or practical reasons, especially in the case of video.

### Challenges of follow-up

The main challenge of follow-up was in locating the participants’ residences given that in most of our research areas, residences lack clear directions, street names, or house numbers. In both urban and rural locations, field workers had to escort the participants to their homes and make a map that they could follow for the next dosing days and home check-ups. Some of the participants preferred to deliver their babies with their mothers in the villages away from their marital homes, which necessitated long-distance travel for field workers’ (sometimes over 150 km) follow-up visits. Additionally, some participants or their family members were uncomfortable with male health workers undertaking follow-up visits and requested female health workers. In these cases, the choices of the participants and their families were respected.

### Summary

Drug development in resource-limited areas presents unique challenges. The means of addressing these challenges must be informed by engagement at multiple levels of the healthcare system. There may be a lack of firm consensus on clinical practice or barriers to accessing standard treatments, where standards are established. Thus there is a need to engage multinational, national, and local healthcare policy and delivery organizations to ensure that studies are designed in a way that reflects evolving clinical practices. The target population may be vulnerable to inducement, which needs to be understood, accounted for, and addressed. Furthermore, local customs, beliefs, and practices should be understood and accounted for in the planning and conduct of the trial.

Clearly, the successful completion of such a research effort relies upon effective partnership with multiple stakeholders, which requires significant effort, investment, creativity, capacity building, and patience. There is a need for awareness of global programs and international and national health policy. Investment needs to be made up front to build capacity and infrastructure in a way that supports the research and is sustainable. There may be substantial effort required on the part of investigator sites to support a large, multicenter pharma industry-sponsored trial aiming to support a stringent regulatory agency submission. Knowledge-sharing between the investigator sites and the Sponsoring Entities helps to ensure that sponsors (especially pharma industry sponsors who may not have an established presence in the geographic research area) learn the local healthcare environment and cultural issues that may impact delivery of healthcare or participation in clinical research. Knowledge-sharing among investigator sites helps to ensure that issues, once identified and understood, are being addressed in a uniform manner or adapted to the local environment, as needed.

Community involvement is essential in effective implementation of clinical trials in Africa. Engagement with family members and study participants is also vital to fully understand their needs during study implementation and to help ensure that study aims are understood in the community. The community’s own leaders are key stakeholders in the community engagement process. Their involvement is important in ensuring that a successful research program is implemented.

## Conclusions

We propose several recommendations based on our experience of conducting malaria trials in rural sub-Saharan Africa. We believe that these recommendations may be applicable to researchers planning to conduct clinical trials in resource-limited settings, assuming that they would face similar challenges. Because one of the Sponsoring Entities for these studies was a large, multinational pharmaceutical company, our recommendations take this into consideration. In each of the recommendations, the industry Sponsoring Entity is encouraged to engage stakeholders at international and national levels in order to understand the background healthcare initiatives and policy and to effectively situate the aims of the research to this context. Sponsoring Entities must also engage local stakeholders with the aim of listening and learning about practices, customs, perceptions, and beliefs of those who will ultimately be contributing to the knowledge generated in the clinical trial. For this reason, it is important for Sponsoring Entities to work closely with local investigators who have a deep understanding of local medical practice and the cultural landscape of the research area. Knowledgeable investigators thus form an indispensable link between the industry Sponsoring Entity and the research participant.

Our recommendations are as follows:

- Engage multinational, national, and local healthcare policy and delivery organizations to ensure that studies are designed in a way that reflects evolving health policy, clinical practices, and regulatory expectations
- Engage investigators and other local healthcare delivery personnel to learn about how health policy is implemented, what traditional beliefs and practices might potentially impact research aims, and how perceptions of healthcare providers might impact patient decisions. With the assistance of investigators, Sponsoring Entities may gain further insights through community engagement:
  - ○ Learn about local traditional beliefs and practices and consider how cultural norms impact the research, ensuring that research protocols acknowledge the context in which they are implemented
  - ○ Inform community leaders about research aims, discussing topics that may have limited local references, such as patient autonomy and the voluntary nature of research participation
- Lastly, Sponsoring Entities and investigators must be aware of potential barriers to informed consent including literacy and laws relating to age of majority

## Abbreviations

AZCQ: Azithromycin-chloroquine
CHMT: Council Health Management Team
EMA: European Medicines Agency
GCP: Good Clinical Practice
IPTp: Intermittent preventive treatment of malaria in pregnancy
MHRA: UK Medicines and Healthcare Products Regulatory Agency
MiP: Malaria in Pregnancy
MMV: Medicines for Malaria Venture
NMCP: National Malaria Control Program
TFDA: Tanzania Food and Drug Authority
WHO: World Health Organization
WHO-GMP: World Health Organization-Global Malaria Program
